# The 2.6 Å Structure of a Tulane Virus Variant with Minor Mutations Leading to Receptor Change

**DOI:** 10.3390/biom14010119

**Published:** 2024-01-16

**Authors:** Chen Sun, Pengwei Huang, Xueyong Xu, Frank S. Vago, Kunpeng Li, Thomas Klose, Xi Jason Jiang, Wen Jiang

**Affiliations:** 1 Department of Biological Sciences, Purdue University, West Lafayette, IN 47907, USAfvago@purdue.edu (F.S.V.);; 2 Division of Infectious Diseases, Cincinnati Children’s Hospital Medical Center, Cincinnati, OH 45229, USA; 3 School of Medicine, Case Western Reserve University, Cleveland, OH 44106, USA

**Keywords:** Tulane virus, histo-blood group antigen, human norovirus, single-particle cryo-EM

## Abstract

Human noroviruses (HuNoVs) are a major cause of acute gastroenteritis, contributing significantly to annual foodborne illness cases. However, studying these viruses has been challenging due to limitations in tissue culture techniques for over four decades. Tulane virus (TV) has emerged as a crucial surrogate for HuNoVs due to its close resemblance in amino acid composition and the availability of a robust cell culture system. Initially isolated from rhesus macaques in 2008, TV represents a novel *Calicivirus* belonging to the *Recovirus* genus. Its significance lies in sharing the same host cell receptor, histo-blood group antigen (HBGA), as HuNoVs. In this study, we introduce, through cryo-electron microscopy (cryo-EM), the structure of a specific TV variant (the 9-6-17 TV) that has notably lost its ability to bind to its receptor, B-type HBGA—a finding confirmed using an enzyme-linked immunosorbent assay (ELISA). These results offer a profound insight into the genetic modifications occurring in TV that are necessary for adaptation to cell culture environments. This research significantly contributes to advancing our understanding of the genetic changes that are pivotal to successful adaptation, shedding light on fundamental aspects of *Calicivirus* evolution.

## 1. Introduction

Human noroviruses (HuNoVs) have been a leading cause of severe gastroenteritis for many years and are still causing many outbreaks all over the world, often with mutations that alter susceptible populations. It is a member of the *Caliciviridae* family, which comprises 11 genera: *Bavovirus*, *Lagovirus*, *Minovirus*, *Nacovirus*, *Nebovirus*, *Norovirus*, *Recovirus*, *Salovirus*, *Valovirus* and *Vesivirus* [1]. All members of the *Caliciviridae* family are nonenveloped viruses exhibiting icosahedral symmetry, with diameters ranging from 27 to 50 nm.

Among the genera in *Caliciviridae*, only *Norovirus* and *Sapovirus* have caused acute gastroenteritis outbreaks in humans. Noroviruses exhibit extensive genetic diversity, encompassing over 10 distinct genogroups. Among these, Genogroup II genotype 4 (GII.4) strain of HuNoV has been responsible for the majority of norovirus outbreaks since 2002. Novel GII.4 variants have continued to be discovered in recent outbreaks and sporadic cases [2]. Research progress on HuNoV was hampered by the lack of a robust cell culture system for more than four decades. Only in 2016 was HuNoV successfully cultured by Dr. Mary Estes and her team at Baylor College of Medicine in stem cell-derived human enteroids with the addition of bile extract [3]. The other genera demonstrate a broad range of animal hosts, spanning from domestic pigs to cats, rabbits, birds, fish and walruses.

**Figure 1:**
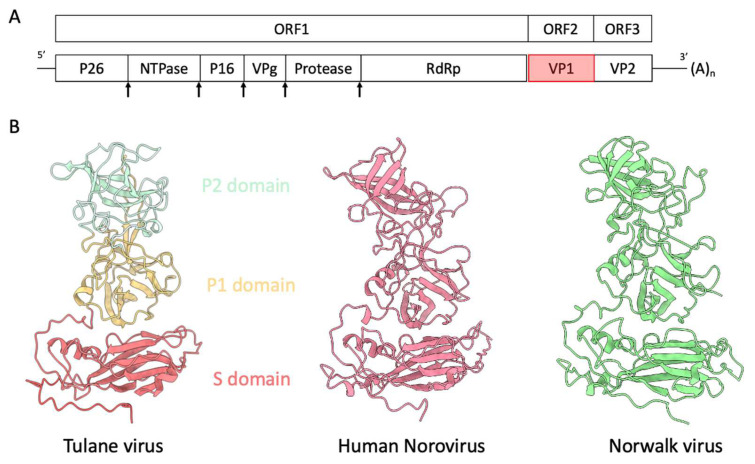
(**A**) Genome organization of Tulane virus. The arrows indicate the putative protease digestion sites. (**B**) Structure comparison of capsid protein VP1 of Tulane virus (PDB: 8VG6), human norovirus VLP (unpublished) and Norwalk virus (PDB: 1IHM). The P1, P2 and S domains of TV VP1 single subunit are colored brown, cyan and coral, respectively.

Considering the lack of an efficient cell culture system for HuNoVs, Tulane virus (TV) [4] stands out as a valuable surrogate for HuNoVs. TV is the prototype of the *Recovirus* genus. It represents a non-segmented, single positive-strand RNA virus with the smallest genome—comprising 6.7 k nucleotides—in the *Caliciviridae* family. Initially isolated from stool samples of Rhesus macaques at the Tulane National Primate Research Center in 2008, TV was found to be cultivable in several monkey kidney cell lines. In comparison to murine norovirus (MNV), TV exhibits a closer genetic and structural affinity to the *Norovirus* genus. TV and HuNoV share similar genome organization (Figure 1A), with three open reading frames: ORF1 encoding a nonstructural polyprotein that undergoes further digestion into several nonstructural proteins, including RNA-dependent RNA polymerase (RdRp); ORF2 encoding the major capsid protein VP1; and ORF3 encoding the minor capsid protein VP2. After binding to its receptor, the VP2 protein of feline calicivirus was observed to assemble into a portal-like structure, potentially serving as a channel for genome translocation [5].

The structure of TV reveals the characteristic T = 3 icosahedral lattice comprising 90 dimers of the capsid protein VP1. Each icosahedral asymmetric unit consists of three subunits—A, B and C—with A and B forming an A/B dimer around the five-fold axes of symmetry, and the C/C dimer around the two-fold axes. VP1 subunits further divide into two domains: the shell domain (S domain), constituting the inner spherical shell of the virus capsid, and the protruding domain (P domain), further divided into the P1 and P2 sub-domains. Notably, the P2 sub-domain is accountable for receptor or antibody binding. VP1, with 534 amino acids and a molecular weight of 57.8 kDa, exhibits high sequence identity across the full length of the capsid protein. Structurally, the capsid protein VP1 of TV, human norovirus (GII.4), and Norwalk virus (Genogroup 1 of *Norovirus*) adopts a similar fold in both the S and P domains (Figure 1B). Within the S domain, an eight-stranded jellyroll fold, a common motif found in virus structures, is observed.

More importantly, it has also been reported to be able to interact with the histo-blood group antigens (HBGAs) [6,7], which are known as the cellular receptor of HuNoVs. HBGAs are carbohydrates found on the cell surfaces in most epithelial tissues and in secretions [8]. HBGAs are also utilized as mediators of infection by many other human pathogens [9], for instance, the rotavirus and some types of bacteria, like *Pseudomonas aeruginosa*, *Helicobacter pylori*, and *enterotoxigenic Escherichia coli* (ETEC).

Previously, the structural study of TV has been hampered by the low titer such that only a few particles can be found under TEM. Therefore, an antibody-based affinity grid method was developed to enrich the virus particles on the EM grid [10,11]. A 2.5 Å resolution TV structure was determined with virus particles captured by the antibody on the grid. However, the virus structure alone does not answer the question of virus–host interactions. In the process of studying the virus receptor complex, a new TV variant (the 9-6-17 strain) was derived in the lab, resulting from adaptation to the cell culture. As confirmed by the results of ELISA, it has lost its binding to the original TV host cell receptor, B-type HBGA. It presents a unique opportunity to study the crucial interactions of TV and receptor binding. Using sequence analysis, structural biology, and mutagenesis, we aim to elucidate the molecular basis of HBGA binding in TV and gain deeper insights into how HuNoVs exploit these receptors to establish infection. Ultimately, our findings may contribute to the development of effective vaccines and antiviral strategies against HuNoVs and other related caliciviruses.

## 2. Materials and Methods

### 2.1. Cell Culture and Purification of Tulane Virus

LLC-MK2 cells were cultured with M199 medium (Thermo Fisher Scientific, Waltham, MA, USA) supplemented with 10% fetal bovine serum, 100 U/mL penicillin, and 100 μg/mL streptomycin. When the 100 mL cells reached a confluency of about 90%, they were inoculated with 300 μL Tulane virus stock (3 × 10^8^ PFU/mL). After 48 h of incubation, the 100 mL infected culture was collected and used to infect 800 mL of LLC-MK2 confluent cell culture. The infection was carried out for 2 h at 37 °C, followed by the replacement of the medium with M199 containing 2% FBS and subsequent incubation at 37 °C. After 48 h of incubation, adherent cells were scraped off and the culture was collected and centrifuged at 2000× *g*. The cell sediments were resuspended and went through three rounds of freeze–thaw cycles to break the cells and release the viruses. Centrifugation at 8000× *g* was performed to remove the cell debris. The supernatant was collected, combined and centrifuged at 150,000× *g* for 2 h with a Ti 50.2 rotor (Beckmann, Brea, CA, USA). The supernatant was discarded, and the sediment was resuspended in PBS pH 7.4 overnight at 4 °C. Then, it was subjected to density gradient centrifugation (Opti-prep; 222,000× *g* for 4 h at 4 °C; SW-41 Ti swinging-bucket rotor) using a Beckman Coulter Optima L-90 ultracentrifuge (Beckman, USA). The gradients were fractionated by bottom puncture. SDS-PAGE electrophoresis was performed to identify the fraction with the major capsid protein VP1 band. The fractions that contained virions were combined and concentrated with a 100 kDa cut-off centrifugal filter unit (Millipore, MA, USA). Finally, the virus sample was buffer-exchanged and stored in PBS at 4 °C.

### 2.2. Viral RNA Extraction and Genome Sequencing

The whole TV RNA genome was extracted with a QIAamp Viral RNA Mini Kit (Cat No./ID: 52904, QIAGEN, Germantown, MD, USA) according to the manufacturer’s protocol. The cDNA library was constructed from the extracted virus RNA using an Illumina TruSeq Stranded Total RNA kit without ribo-depletion. The cDNA library was sequenced using a MiSeq (Illumina, CA, USA) in the Purdue Genomics Core Facility. The genome was assembled from raw data using the SPAdes software v3.0.0. The wild-type TV sequence was obtained from GenBank: EU391643.1. The genome sequence of this 9-6-17 TV will be deposited to GenBank.

### 2.3. Saliva-Based ELISA for Measurement of HBGA Binding

The saliva samples were treated via boiling for 10 min prior to the assay for the denaturation of potential antibodies that may interfere with the assay. The expression levels of the A, B, H-type 1, H-type 2, and Lewis’s antigens in the saliva were determined previously using anti-H type 1 (BG-4), anti-Leb (BG-6), and anti-Ley (BG-8) (Signet Laboratories Inc. Dedham, MA, USA) MAbs, and anti-H type 2 (BCR 9031), anti-A (BCR 9010), and anti-B (BCRM 11007) MAbs (Accurate Chemical and Scientific Corporation, Westbury, NY, USA). To test HBGA binding by the TV strains, saliva samples diluted with PBS at 1:1000 were coated onto plates at 4 °C overnight, and then, incubated with a serially diluted TV preparation. The salivary HBGA-bound TVs were detected by mouse anti-TV serum (1:3500), and then, by HRP-conjugated goat anti-mouse IgG (1:5000). The color signal was developed with the TMB (3,3′,5,5′-Tetramethylbenzidine), and OD was read at a wavelength of 450 nm.

### 2.4. Cryo-EM Sample Grid Preparation and Data Acquisition

Purified 9-6-17 TV virions were applied onto R1.2/1.3 300 mesh graphene oxide-coated Quantifoil grids (Ted Pella Inc., Redding, CA, USA) or 300 mesh graphene oxide-coated lacey carbon grids (Ted Pella Inc., Redding, CA, USA), and then, plunge-frozen with a Cryoplunge 3 system (Gatan, Pleasanton, CA, USA). The frozen sample grids were imaged on a 300 kV Titan Krios electron microscope (Thermo Fisher Scientific, Waltham, MA, USA) equipped with a post-GIF K2 summit camera mounted on a 20 eV slit Quantum energy filter (Gatan, Pleasanton, CA, USA) in super-resolution mode. Three datasets were collected for the 9-6-17 TV: 5-12-18 dataset, without DTT retreatment and with DTT treatment. Details of the data collection conditions are listed in Table S1.

### 2.5. Image Processing

The movies were aligned with Motioncor2/1.0.5 [12] and binned to 1.5×. The dataset with DTT treatment was binned to 1.6×. Subsequently, CTF determination of dose-weighted micrographs was performed with CTFFIND4 [13]. The particles were picked via cisTEM [14] and imported into cryoSPARC [15] for 2D classification, ab-initial reconstruction and homogeneous refinement with icosahedral symmetry. The particle numbers retained in each step are listed in Table S1. The cryoSPARC homogenous refined maps were further refined with icosahedral symmetry in JSPR [16,17]. The viral RNA genome exhibits notably strong intensity. To mitigate the influence of RNA density on the FSC calculation, a circular mask with a diameter of 210 Å was utilized to exclude the inner RNA density. Subsequently, a larger circular mask, 420 Å in diameter, was applied to envelop the entire virus before employing the *trueFSC.py* program in JSPR. This program generates an adaptive mask based on the mass of the viral protein shell. The local resolutions were evaluated using RELION/3.0 [18,19,20,21,22]. The maps were sharpened with EMAN2 [23] *e2proc3d.py* or DeepEMhancer [24].

### 2.6. Model Refinement

The PDB model of the wild-type TV structure (PDB: 8VG6) was fitted into the cryo-EM maps of the 9-6-17 TV with *Chimera* [25]. The asymmetric unit was cut out from the entire virus map and refined with *phenix.real_space_refine* [26] and *Rosetta* [27] via the GNU parallel system [28]. The model with the best model-map FSC score was selected for further analysis. All models and maps were visualized in *Coot* [29]. The data collection and model statistics are shown in Table S1.

## 3. Results

### 3.1. A New TV Variant Has Lost Its Ability to Bind to the Type B HBGA Receptor

**Figure 2:**
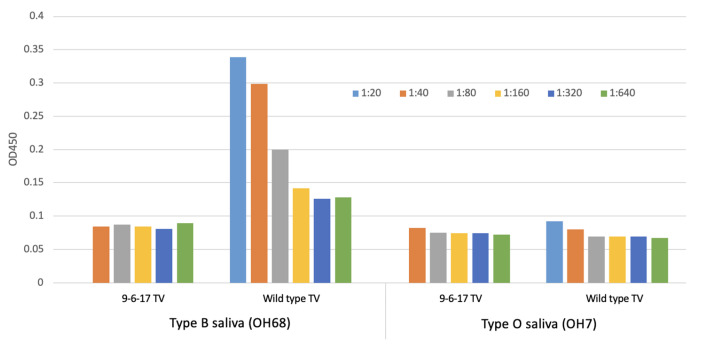
Saliva-based ELISA for measurement of HBGA binding of TV and the growth curve of the 9-6-17 TV variant. One type B saliva sample (OH68) and one type O saliva sample as a negative control were used for testing the change in TV binding to the blood type B antigen. The titer of the 9-6-17 TV was 10^9^ PFU/mL. The initial concentration in the ELISA starting at 1:40 was 2.5 × 10^7^ PFU/mL. The wild-type TV sample was from the PBS-dialyzed pool of wild-type TV peak fractions (F7 and F8) after CsCl density gradient centrifugation. The wild-type TV sample was prepared in 2016 and the virus titer was estimated to be 3 × 10^8^ PFU/mL based on the plaque assay. The virus inoculum was generated in 2015. The initial concentration in the ELISA starting at 1:20 was 1.5 × 10^7^ PFU/mL.

In the process of TV culture, we obtained a new TV variant that has lost the ability to bind to its receptor, HBGA. We named this variant the 9-6-17 TV. We purified the 9-6-17 TV and performed ELISA with the type B saliva sample (OH68) and type O saliva sample (OH7), representing B-type and O-type HBGA. It has been shown previously that Tulane virus can be adhered to the type B saliva samples [30]. The type O saliva sample was used as a negative control since it has been reported to be the phenotype that TV does not interact with. Different types of saliva samples were first applied to the plate, and the serial diluted virus samples were added subsequently to measure the binding ability. The absorbance signal should correspond to the amount of virus that binds with the saliva sample on the plate. Figure 2 demonstrates distinct A450 absorbance patterns in the wild-type TV and the 9-6-17 TV when exposed to the type B saliva sample. The wild-type TV exhibits a robust A450 signal with a clear dose–response relationship, displaying a decrease in absorbance as the virus sample concentration decreases. Conversely, the 9-6-17 TV presents a notably weak absorbance signal, regardless of the concentration of the virus sample. These findings suggest significant divergence between the wild-type TV and the 9-6-17 TV in their responsiveness to the type B saliva sample.

### 3.2. Sequence Analysis of the 9-6-17 TV Strain

**Figure 3:**
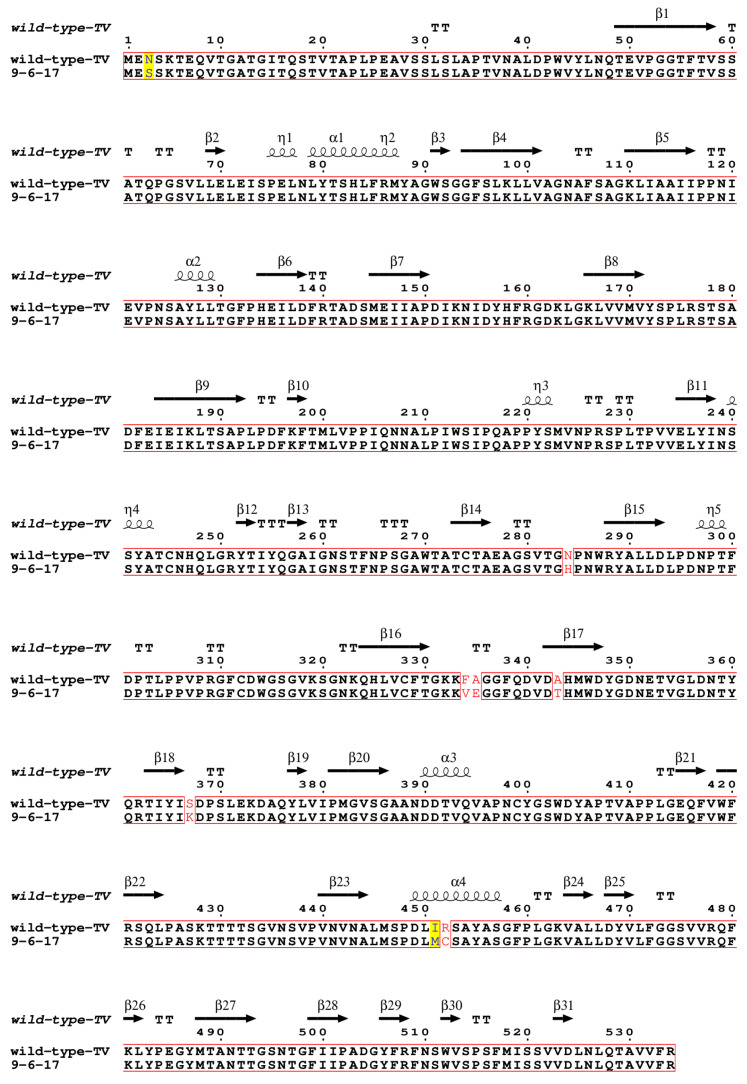
Sequence alignment of VP1 of the wild-type TV and the 9-6-17 TV strains. The wild-type TV sequence was obtained from GenBank under accession number EU391643. The GenBank accession number of 9-6-17 TV is PP098449. The sequence alignment was performed using Clustal Omega [31,32,33] and displayed using ESPript 3.0 [34]. The identical residues were marked in red boxes. Residues with high similarities were highlighted with a yellow background, while those with low similarities were colored in red.

To investigate the genetic difference in this new TV strain from the wild-type TV, we performed whole-genome sequencing with the extracted viral RNA of the purified virions. Compared to the wild-type TV (GenBank: EU391643), the first eight nucleotides at the 5′ end of the 9-6-17 TV genome are missing, and 31 nucleotide mutations are identified in the 9-6-17 TV, which is about 0.45% of the whole genome length (Table 1). Among the eighteen amino acid mutations, eight amino acid mutations were located in the major capsid protein VP1, which is responsible for the receptor binding in the infection process. The eight amino acid mutations in VP1 are N3S, N284H, F334V, A335E, A343T, S367K, I451M and R452C. The multi-sequence alignment of the 9-6-17 TV strain and the wild-type TV capsid protein VP1 is shown in Figure 3.

We next assessed which of these mutations are adaptive mutations instead of random mutations. We examined the VP1 sequence of ten other *Recovirus* strains. We compared the amino acid identity at each mutation site in VP1 in these *Recovirus* strains and listed them in Table S2. Among the eight mutation sites, only A343T is a conservative substitution. Nine of the homolog proteins have alanine at position 343, but there is one homolog protein that has threonine. For position 3 of VP1, six homolog proteins in the same genus have the same amino acid serine as in the 9-6-17 TV instead of asparagine in the wild-type TV. This suggests that this mutation in 9-6-17 TV is probably evolutionarily favored over the residue asparagine at position 3 since it is more frequently found in other *Recovirus* strains. The same situation applies to N284H and F334V. For the N284H mutation, it has a structural basis whereby it can form a π–π stacking interaction with tryptophan at position 287, as shown in Figure S1.

At position 35, all ten *Recovirus* sequences are aspartic acid, while the 9-6-17 TV has a glutamic acid replacing alanine at that position. The 335 position is at the tip of the P domain. It is near the dimer interface but is not directly involved. The side chain of Asp335 protrudes outward and does not interact with neighboring residues. Therefore, this residue is very likely to be involved in receptor binding. Since both aspartic acid and glutamic acid are negatively charged, it must be essential to have negatively charged residues at this position.

Among the eight mutations, only the S367K and the conjunct mutations I451M and M452C are unique in our 9-6-17 TV strain. They are not observed in any other *Recoviruses*. However, the S367K mutation is less likely to be a random mutation because the S367K mutation is induced by two nucleotide changes, from codons AGT to AAA. The probability of two consecutive random mutations should be minimal, although we could not rule out this possibility.

### 3.3. Structure Determination of the 9-6-17 TV

**Figure 4:**
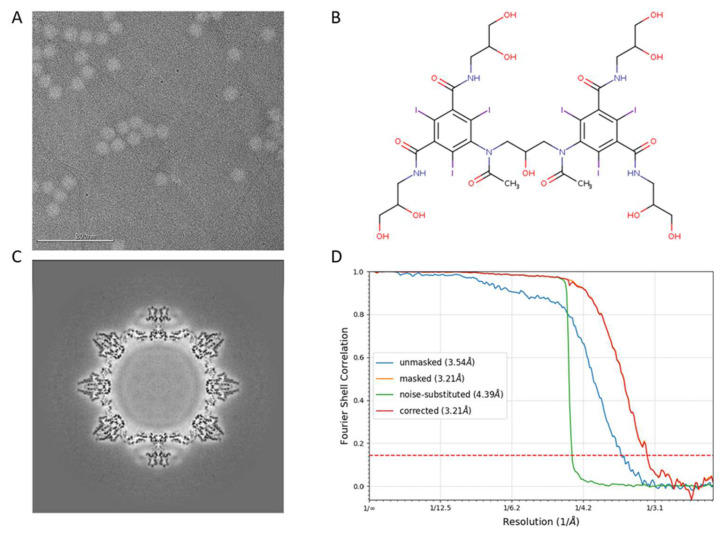
The first dataset of 9-6-17 TV. (**A**) The representative micrograph shows the opposite contrast due to the iodixanol in the background. The scale bar is 200 nm. (**B**) The chemical structure of iodixanol (OptiPrep). (**C**) The central section of the reconstructed map before contrast inversion. (**D**) The FSC curve of the final reconstruction. The red dotted line is where FSC equals 0.143.

We have previously determined the structure of wild-type TV (PDB:8VG6) to a 2.6 Å resolution using antibody-based affinity cryo-EM [10]. To investigate the structural variation in the 9-6-TV, we collected three cryo-EM datasets of the purified 9-6-17 TV. The first dataset that we collected for 9-6-17 TV has inverted image contrast whereby the virus particle is brighter than the background (Figure 4A). We reasoned that this was likely caused by the residue iodixanol (OptiPrep) in the buffer not being fully dialyzed away. Iodixanol is the density gradient that we used in the final purification step. It has several iodine atoms in its structure (Figure 4B) that are able to scatter electrons stronger than virus particles. Therefore, the residual iodixanol in the buffer created a darker background in the cryo-EM image. Despite the reverse contrast, the reconstruction was performed as normal, and the map contrast can be easily inverted by multiplying −1 with the map voxel values (Figure 4C). In the end, a 3.2 Å resolution map (Figure 4D) was generated and the atomic model was derived from it.

### 3.4. Disulfide Bond from the R452C Mutation Stabilizes Dimer Interaction

**Figure 5:**
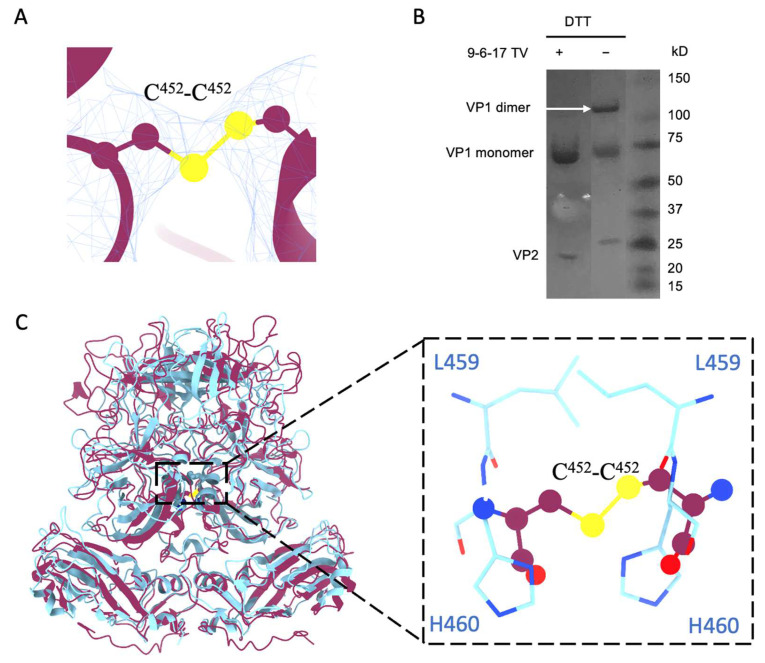
The disulfide bond formed by Cys^452^-Cys^452^. (**A**) The density of the disulfide bond of Cys^452^ at the dimer interface. The contour level of the map for TV without DTT in ChimeraX is set to 2. The sulfur atoms are depicted in yellow, the electron density map is represented by a blue mesh, and the TV model is presented in purple. (**B**) The SDS-PAGE result of 9-6-17 TV with or without DTT treatment. Without reducing agents, the 9-6-17 TV shows a dimer band at around the 120kD position, while the one with reducing agents does not have the dimer band. (**C**) Superimposition of norovirus VLP (PDB:7MRY) VP1 dimer (cyan) onto TV VP1 dimer (purple). Note that the position of the Cys^452^-Cys^452^ disulfide bond aligns precisely with the location where His^460^ and Leu^459^ interact with the Cd^2+^ ion in the human norovirus VLP structure. The flattened circles represent the Cys^452^-Cys^452^ disulfide bond. The Cyan residues belong to norovirus VP1.

When we examined the density map of the 9-6-17 TV, we found a density bridge connecting the two subunits of A/B and C/C dimers at the position of Cys^452^ (Figure 5A). This density is consistent with the Cys^452^-Cys^452^ disulfide bond within all the dimers. To verify the dimerization of VP1 bridged by the disulfide bond, we performed SDS-PAGE with and without reducing reagents (DTT and beta-ME) in the loading buffer. A band at around a 120 kDa molecular weight, the mass of a VP1 dimer, was observed in the sample without DTT but not in the sample with DTT (Figure 5B). This indicates that the Cys^452^-Cys^452^ disulfide bond covalently linked the A/B or C/C subunits in a dimer. Research has shown that the Cd^2+^ metal ion is bound between the P dimers via a conserved residue, His^460^, to stabilize the P dimer conformation in human norovirus GII.4 [35]. The His^460^-Cd^2+^ -His^460^ interaction site is in exactly the same position as the disulfide bond in TV, as shown in Figure 5C. This indicates an evolutionary preference for stabilizing P dimers at the center position for *Caliciviruses*.

**Figure 6:**
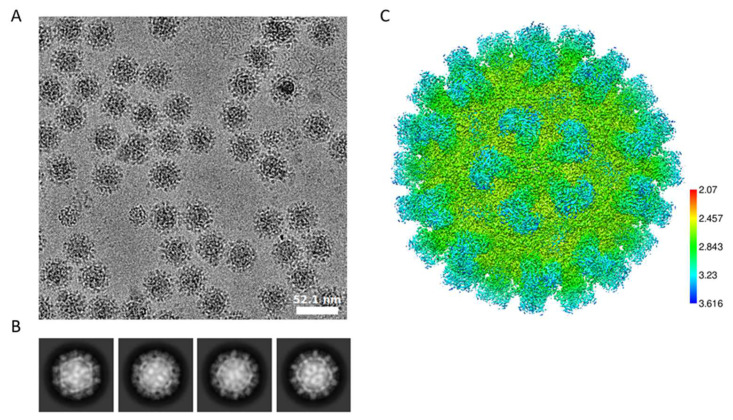
The 9-6-17 TV cryo-EM dataset in which the virus sample was not treated with DTT. (**A**) Representative image without low-pass filter showing high-contrast virus particles. The scale bar is 52.1 nm. (**B**) The 2D class averages show different virus orientations with clear spikes on the virus surface. (**C**) Local resolution of the map reconstructed from this dataset.

Two additional datasets were then collected to further explore the effects of the Cys^452^-Cys^452^ disulfide bond in the virus structure. To reduce this disulfide bond, we treated one sample with DTT and the other one without DTT to serve as the control sample. These two datasets used the same batch of viruses and were collected in the same session. The OptiPrep was largely dialyzed away for this sample. These two datasets have normal image contrast and 2D class averages, as shown in Figure 6A–C, which indicates that the final reconstructed map has an average local resolution around 2.8 Å for the shell and only slightly lower resolution for the spikes.

**Figure 7:**
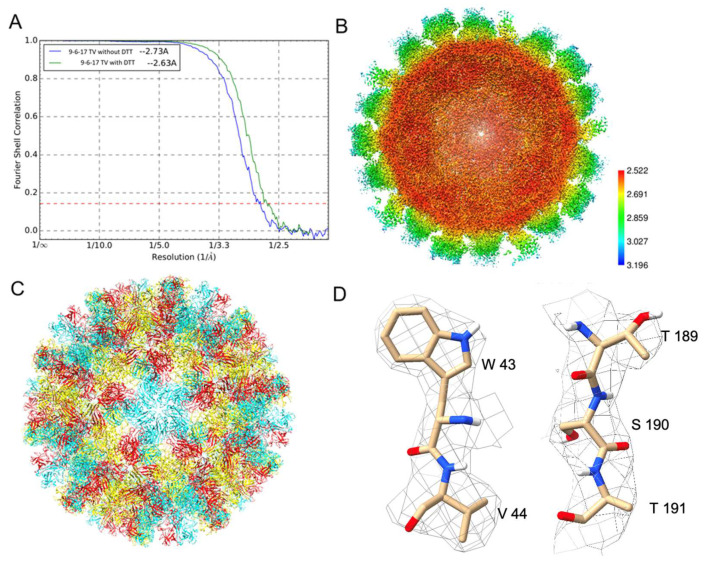
The 9-6-17 TV datasets with and without DTT treatment. (**A**) The gold-standard Fourier shell correlation (FSC) curves of these two datasets. (**B**) The cross-section of the reconstructed map with DTT treatment shows the estimated local resolution. (**C**) The ribbon diagram of the full capsid shows the T = 3 icosahedral organization with subunit A (blue), subunit B (red) and subunit C (yellow) colored, respectively. (**D**) Two segments, a.a. 43–44 and a.a. 189–191, were selected to present the quality of the electron density map. The electron density map is overlaid on the final refined structure of subunit A. The contour level of the DeepEMhancer sharpened density map is 0.138.

The datasets with and without DTT reached 2.63 and 2.73 Å resolutions, respectively, represented by the gold standard Fourier Shell Correlation (FSC) curves in Figure 7A. The local resolution of the map without DTT indicates a near 2.5 Å resolution for the shell region and around a 2.8 Å resolution for the P domain in Figure 7B. Density-guided real-space refinement of the TV capsid protein VP1 model into the reconstructed maps was performed using the programs COOT and phenix. The refined model of asymmetric units was assembled into a full virus model in Figure 7C. Each asymmetric unit consists of three VP1 monomers, named subunits A, B and C. Two representative volumes of density superimposed with the atomic model are displayed in Figure 7D. Compared to the map from the dataset without DTT, the DTT-treated map has a large bulb of extra density at the top of the P domain near residues His284 and Pro285, as shown in Figure S2. The identity of this extra density is unknown.

**Figure 8:**
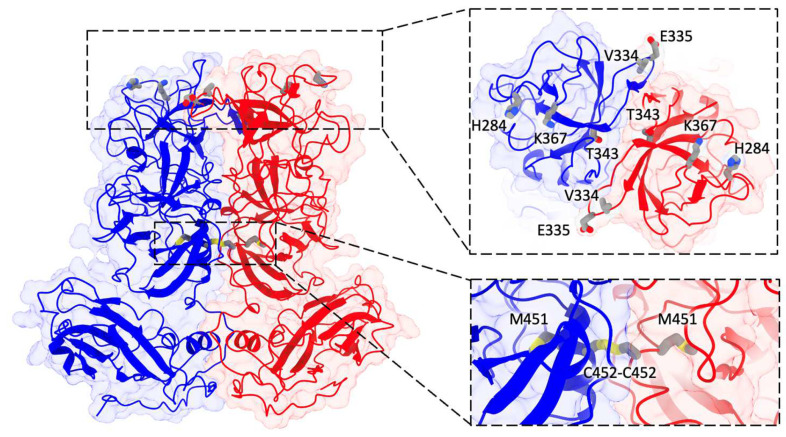
The atomic model of the 9-6-17 TV DTT-treated map. Subunit A is colored blue, while subunit B is colored red. A refined model of the AB dimer with seven of the eight mutation sites (except for N3S) is displayed and labeled. The insertion on the right shows the 90-degree tilted view of the top of the P domain containing 5 mutation sites. The bottom insert shows a close-up view of the two mutations at the dimer interface.

Our primary focus lies in the identification of mutation sites. In Figure 8, we labeled all the mutated residues within the refined 9-6-17 TV model. Notably, four of these mutations are situated on the top surface of the P2 domain, which serves as the interaction surface with host cell receptors. Additionally, two mutations (I451M and R452C) are positioned at the interface of the P dimer, contributing to the stabilization of the P dimer through the formation of a disulfide bond.

### 3.5. Elongated Extra Density in the Hydrophobic Pocket in the P Dimer

**Figure 9:**
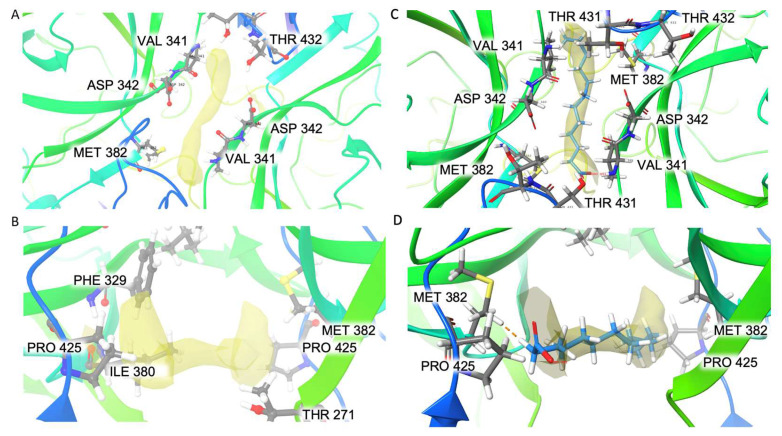
Extra density in the hydrophobic pocket of the two dimers shown in top view (**A**) and side view (**B**). Lauric acid is fitted into the extra density: top view (**C**), side view (**D**).

An elongated extra density (Figure 9A,B) was found at the interface of the two subunits in both the A/B dimers and the C/C dimers near residues P425, V341 and M352. It has a volume of ~94 Å^3^ and a surface area of ~220 Å^2^. The pocket around it is mostly composed of hydrophobic residues (F329, V341, I380, M382, P425) with only one charged residue, D342, and one polar residue, T432. Picornaviruses are known to have a pocket factor in the five-fold canyon to stabilize the virus capsid, and once the virus binds to its receptor, the pocket factor is dislodged, which induces genome release [36]. However, a pocket factor has not been reported in *Caliciviruses*. The pocket factor of human enterovirus 71 (EV71) was modeled as lauric acid. We tried to fit the unmodified lauric acid (C_12_H_24_O_2_) into the extra density (Figure 9C,D). It has the same length of extra density, but the extra density appears to have C2 symmetry, while the lauric acid does not have symmetry. Since this extra density exists in both the A/B (C2 symmetry not imposed) and C/C (C2 symmetry imposed) dimers, its C2 symmetry reflects the true shape of an unknown molecule instead of an artifact of image processing.

## 4. Discussion

Histo-blood group antigens (HBGAs) are intricate terminal carbohydrates found on cellular surfaces and are also secreted into various bodily fluids, including the saliva and intestinal secretions. The synthesis of these carbohydrates originates from disaccharide precursors, undergoing a stepwise addition of monosaccharides through the activity of specific glycosyltransferases in defined locations, ultimately determining the distinct types of HBGAs. They are widely distributed on red blood cells and mucosal epithelium cells, playing a crucial role in human susceptibility to various virus infections, including HuNoVs and rotavirus. TV, a non-pathogenic surrogate for HuNoVs, has also been reported to utilize HBGAs as receptors.

Here, we introduce the novel 9-6-17 TV variant, showcasing distinct alterations from the wild-type TV. Previous studies demonstrated TV’s recognition of B-type HBGAs and specific groups of A-type HBGAs. However, our findings reveal a remarkable loss of binding ability in the 9-6-17 TV variant toward its original receptor, B-type HBGA, resembling observations in certain norovirus strains that don’t bind to HBGAs [37].

Intriguingly, we observed additional density in the hydrophobic pocket between the P dimers, which is at the exact same location as that in which murine norovirus binds to the bile acids GCDCA (glycochenodeoxycholic acid) and LCA (lithocholic acid) [38]. While bile acids act as a cofactor for murine norovirus infection and are essential for human norovirus infection [3], TV, in contrast, does not require bile acid supplementation. This raises the prospect of an unidentified ligand in the hydrophobic pocket, warranting further exploration for future studies.

These outcomes suggest potential alternative receptors for both TV and HuNoVs. Despite strong evidence linking HBGAs to human norovirus infection, reports hint at the existence of other co-factors or receptors, at least for specific strains. Our study underscores the importance of specific amino acid residues in HBGA recognition, offering insights for developing broadly neutralizing antibodies or antiviral compounds targeting these interaction sites. Understanding the diversity of potential receptors used by *Caliciviruses* can inform the design of multivalent vaccines, providing broader protection against various viral strains and genotypes.

While our study presents a detailed analysis of the 9-6-17 TV variant, it highlights the need for further investigations. Future studies are needed to identify the ligand identity in the hydrophobic pocket and confirm its functional significance. Additionally, higher-resolution structural analysis without icosahedral symmetry could unveil the finer details of the ligand density.

In sum, our study elucidates the pivotal role of receptor adaptation in TV, by presenting a novel TV variant that lacks the binding ability to its receptor while remaining infectious and identifying critical residues responsible for the loss of receptor binding ability. These findings pave the way for ongoing research into alternative receptors and co-factors of human norovirus, contributing to the development of effective preventative and therapeutic strategies against it.

## Figures and Tables

**Table 1:** Statistics of the 9-6-17 TV sequence.

	9-6-17 TV
Sequence length (bp)	6701
Number of nucleotide mutations in total	31
Number of nucleotide mutations in ORF2	9
Number of nucleotide mutations in ORF3	7
Number of amino acid mutations in total	18
Number of amino acid mutations in VP1	8
Number of amino acid mutations in VP2	5

## Data Availability

The 9-6-17 TV DNA sequence has been deposited to GenBank with the accession ID of PP098449. The atomic model of the first 9-6-17 TV dataset, the 9-6-17 TV without DTT treatment dataset and the 9-6-17 TV with DTT retreatment dataset have been deposited to PDB with the accession codes 8VGR, 8VJS and 8VJR. Their reconstructed maps have been deposited to EMDB with the accession codes EMD-43222, EMD-43293 and EMD-43292.

## Supplementary Materials

The following supporting information can be downloaded at: https://www.mdpi.com/article/10.3390/biom14010119/s1, Table S1: Cryo-EM data collection and refinement statistics; Table S2: Residue identity at the 8 mutation sites in VP1 of 10 *Recovirus* homologues in *Calicivirus* family; Figure S1: The interaction of H284 and W287 in the 9-6-17 TV structure; Figure S2: Extra density near TV VP1 subunits A His284 and Pro285. It is observed on all subunits. The contour level of the TV DTT-treated map is set to 8.58 in UCSF ChimeraX-1.6.
