# Supplementary material for: The 2.6 Å Structure of a Tulane Virus Variant with Minor Mutations Leading to Receptor Change
Source: Biomolecules. 2024 Jan 16;14(1):119. doi: 10.3390/biom14010119 (PMC10813083; doi:10.3390/biom14010119)
Supplement: Supplementary file 1 [file biomolecules-14-00119-s001.zip › supplementfigures.pptx]

## Slide 1
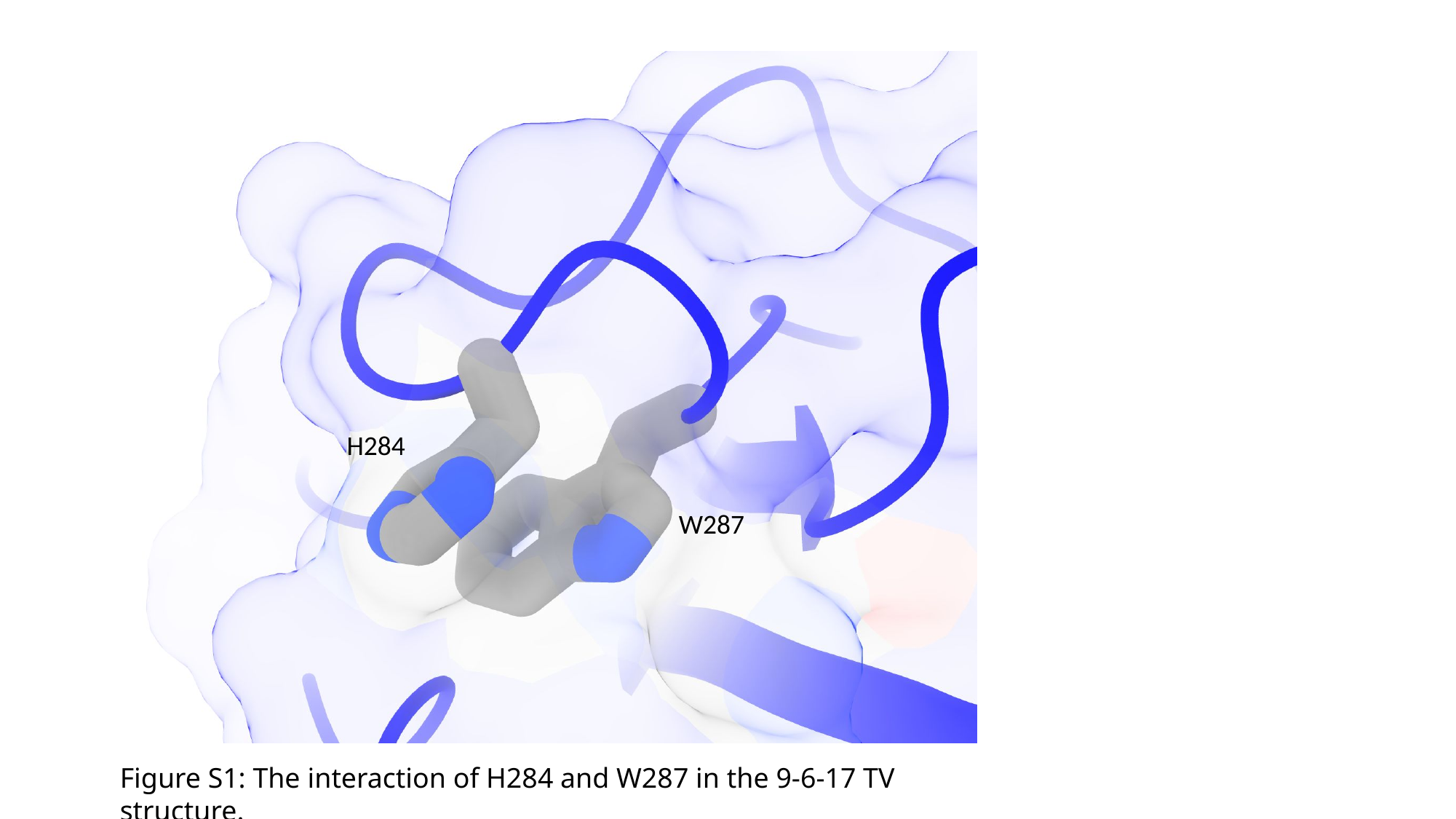

H284
W287
Figure S1: The interaction of H284 and W287 in the 9-6-17 TV structure.

## Slide 2
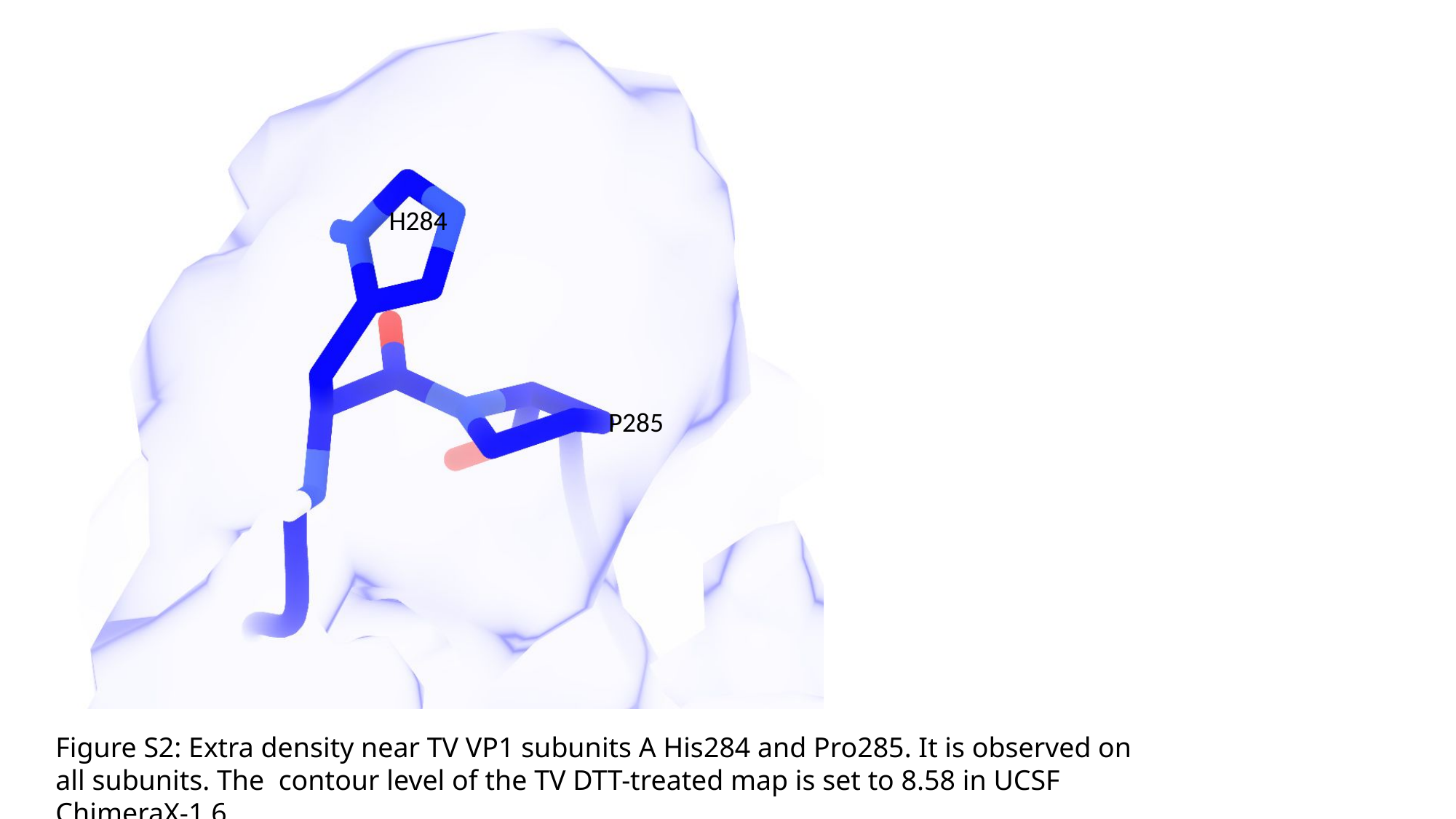

H284
P285
Figure S2: Extra density near TV VP1 subunits A His284 and Pro285. It is observed on all subunits. The contour level of the TV DTT-treated map is set to 8.58 in UCSF ChimeraX-1.6
