# Supplementary material for: The 2.6 Å Structure of a Tulane Virus Variant with Minor Mutations Leading to Receptor Change
Source: Biomolecules. 2024 Jan 16;14(1):119. doi: 10.3390/biom14010119 (PMC10813083; doi:10.3390/biom14010119)
Supplement: Supplementary file 1 [file biomolecules-14-00119-s001.zip › Table-S1-S2.pdf]

Table S1. Cryo-EM data collection and refinement statistics.

Sample	9-6-17 TV 5-12-18 dataset	9-6-17 TV Without DTT	9-6-17 TV With DTT
**Data collection**			
Grid	Lacey grid coated with graphene oxide, 300 mesh	Quantifoil grid coated with graphene oxide R1.2/1.3 300 mesh	
Voltage (kV)	300		
Total Dose (e$^-$/Å^2)	23	25	
Nominal Magnification	81,000	105,000	
Number of Movies	969	503	771
Physical Pixel Size (Å)	1.73	1.384	
Intended Defocus (μm)	0.8-2.2	1.5-2.5	
Number of Frames	35	30	
**Image processing**			
Particles Picked	48,505	20,260	132,461
Number of Particles After 2D classification	27,187	18,397	44,239
Final number of particles	11,645	16,777	44,239
Resolution (Å)	3.2	2.73	2.63
Final Map Pixel Size (Å)	1.2975	1.035	1.104
**Refinement**			
Map CC	0.73	0.81	0.85
All-atom Clashscore	15.25	6.79	8.88
Rotamer Outliers (%)	4.90	2.3	1.99
**Ramachandran plot**			
Outliers (%)	0.20	0.26	0.33
Allowed (%)	11.27	9.71	11.84
Favored (%)	88.53	90.03	87.84

Table S2. Residue identity at the 8 mutation sites in VP1 of 10 Recovirus homologues in *Calicivirus* family

a.a. position	a.a. in wild-type TV	a.a. in 9-6-17 TV	10 Recoviruses capsid protein Uniprot Entry ID

3	N	S	S(AHE37931.1;AHE37934.1;AHE37928.1;AHE37919.1; AHE37916.1; AHE37913.1),<br><br>K(AHE37922.1;AFV48067.1;AHE37925.1;AHE37947.1)
284	N	H	H(AHE37919.1; AHE37916.1; AHE37913.1),<br><br>Q(AHE37925.1; AHE37947.1; AHE37931.1; AHE37934.1; AHE37928.1),<br><br>N(AFV48067.1),<br><br>K(AHE37922.1)
334	F	V	V(AHE37922.1;AFV48067.1;AHE37925.1;AHE37947.1;AHE37919.1;AHE37916.1;AHE37913.1),<br><br>T(AHE37931.1; AHE37934.1; AHE37928.1)
335	A	E	D(AHE37922.1; AFV48067.1; AHE37925.1; AHE37947.1; AHE37919.1; AHE37916.1; AHE37913.1; AHE37931.1; AHE37934.1; AHE37928.1)
343	A	T	A(AHE37922.1; AFV48067.1; AHE37925.1; AHE37947.1; AHE37916.1; AHE37913.1; AHE37931.1; AHE37934.1; AHE37928.1),<br><br>T (AHE37919.1)
367	S	K	S(AHE37919.1; AHE37916.1; AHE37913.1; AHE37931.1; AHE37934.1; AHE37928.1),<br><br>I(AHE37922.1; AFV48067.1; AHE37925.1; AHE37947.1)
451	I	M	I(AHE37922.1; AFV48067.1; AHE37925.1; AHE37947.1; AHE37919.1; AHE37916.1; AHE37913.1; AHE37931.1; AHE37934.1; AHE37928.1)
452	R	C	R(AHE37922.1; AFV48067.1; AHE37925.1; AHE37947.1; AHE37919.1; AHE37916.1; AHE37913.1; AHE37931.1; AHE37934.1; AHE37928.1)

*References: (AHE37922.1; AHE37925.1; AHE37947.1; AHE37919.1; AHE37916.1; AHE37913.1; AHE37931.1; AHE37934.1; AHE37928.1) [27]; AFV48067.1 [28].
